# Effects of heart rate on cardiac function in normal mice and rats and in animal models of heart failure

**DOI:** 10.3389/fphys.2026.1847051

**Published:** 2026-06-16

**Authors:** Jiuli Zhao, Xiang Ji, Qing Xu, Shaojiao Liu, Sha Su, Yu Teng, Lei Wang, Mingjing Zhao

**Affiliations:** 1 Key Laboratory of Chinese Internal Medicine of Ministry of Education, Dongzhimen Hospital Affiliated to Beijing University of Chinese Medicine, Beijing, China; 2 Capital Medical University, Core Facilities Centre, Beijing, China

**Keywords:** animal experiment, cardiac imaging, echocardiography, heart failure, heart rate

## Abstract

**Objectives:**

To identify the practical heart rate measurement windows by examining the impact of various heart rate ranges on changes in cardiac function in small animal models of health and disease under isoflurane anesthesia using cardiac ultrasonography.

**Methods:**

Male Sprague–Dawley rats were used to produce a model of post-myocardial infarction heart failure by left anterior descending artery ligation. Each of the model and normal (control) groups included 11 rats. On days 7 and 28 after surgery, the model group showed dynamic alterations in cardiac function. A “dual-hit” strategy combining nitric oxide synthase inhibitors with high-fat diet was used to create a mouse model of heart failure with intact ejection fraction. In addition to comparing failed and normal hearts at the same heart rate loads, echocardiography was used to evaluate cardiac performance in normal and model rats and mice under various heart rate loads controlled by varying depths of anesthesia.

**Results:**

Under normal or pathological conditions, changes in heart rate had a minor impact on contractile performance; however, when the heart rate was reduced, the contractile function was markedly decreased. Both rats and mice exhibited diastolic dysfunction when their heart rates decreased.

**Conclusions:**

Echocardiographic systolic function in rats is not significantly impacted by changes in heart rate under normal or pathological conditions. In contrast, bradycardia under isoflurane anesthesia easily causes diastolic dysfunction in both species, and systolic performance in mice decreases as the heart rate is reduced. Thus, the practical heart rate measurement windows for echocardiography under isoflurane anesthesia may be 390–450 bpm for rats and 450–480 and 510–550 bpm for mice.

## Graphical Abstract

**Figure:**
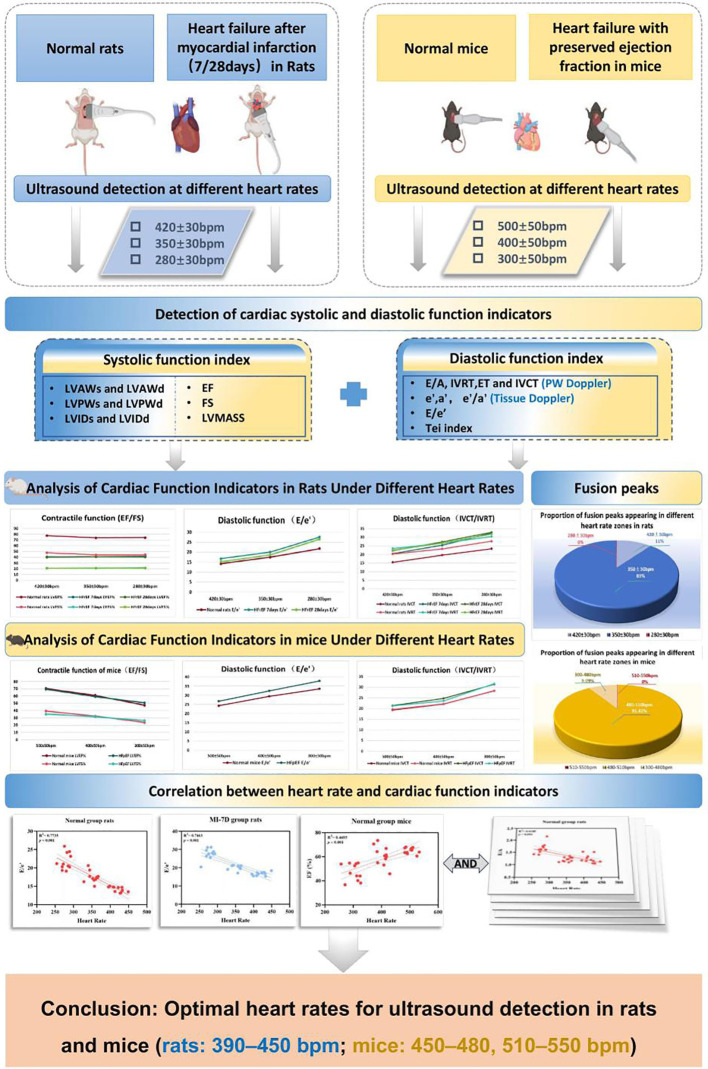
A, late diastolic peak Doppler blood flow velocity; EF, left ventricular ejection fraction; e’, peak tissue Doppler velocity of myocardial relaxation at the mitral annulus during early diastole; E, the peak early Doppler blood flow velocityat the mitral valve; FS, left ventricular short-axis shortening fraction; IVRT, isovolumetric relaxation time; IVCT, isovolumetric contraction time; LVAWs, left ventricular end-systolic anterior wall thickness; LVAWd, left ventricular end-diastolic anterior wall thickness; LVPWs, left ventricular end-systolic posterior wall thickness; LVPWd, left ventricular end-diastolic posterior wall thickness; LVIDs, left ventricular end-systolic internal diameter; LVIDd, left ventricular end-diastolic internal diameter.

## 1 Introduction

Echocardiography has emerged as the “gold standard” diagnostic method for evaluating the structure and function of the heart in clinical cardiology due to its affordability, ease of use, speed, reproducibility, and non-invasiveness. Its use in preclinical small animal research is also expanding, providing a key means for monitoring the dynamic changes in cardiac structure and function in various cardiac disease models (Salerno et al., 2025). Heart rate is one of the intrinsic regulatory factors of cardiac output within the body. The diastolic phase (particularly the rapid filling phase) is significantly shortened when the heart rate rises, which usually has a detrimental impact on diastolic function. Increased heart rate often has a negative effect on diastolic function, resulting in inadequate left ventricular filling time and a marked shortening of the diastolic period, particularly the rapid filling period. Clinically, in the event that the heart rate increases considerably and the early diastolic flow velocity (E peak) of the mitral valve drops to <20 cm/s before the onset of atrial contraction, the A peak of the mitral valve rises and the E/A ratio falls. A false positive result of left ventricular relaxation impairment could arise from the merger of early and late diastolic spectra, which would provide an E/A ratio <1 (Nagueh et al., 2025).

Furthermore, research revealed a substantial association between the longitudinal strain and strain rate of the mouse myocardium in low heart rate states, and the longitudinal strain and strain rate were significantly lower in low heart rate states compared with waking heart rate and high heart rate states (Castiglioni et al., 2015). An increase in heart rate within a specific range can have a positive inotropic impact by increasing myocardial contractility through calcium ion inflow and calcium release from the sarcoplasmic reticulum per unit time. Frank–Starling’s law, however, states that when an increase in heart rate is not enough to offset a decrease in stroke volume, cardiac output may actually stop increasing or even start to decrease, thereby impairing normal contractile function, as Yu et al. discovered in their study of isolated rat hearts (Haroon et al., 2021). Both abnormally high and excessively low heart rates can produce false positive or negative results in investigations, jeopardizing their scientific validity. Consequently, during small animal cardiac ultrasound examinations, a steady heart rate range suited for imaging is a critical requirement for maintaining data quality and the dependability of study findings. Previous research and practice showed that in small animal experiments, the heart rate of anaesthetized ill animals is markedly reduced. Heart rate is more important than anesthetic duration (Weytjens et al., 2009; Wu et al., 2010). Researchers have also investigated the effects of several anesthetic drugs on heart function in mice (Pachon et al., 2015; Li et al., 2019). However, no comprehensive study has evaluated the impact of heart rate fluctuations on cardiac function in healthy small animals or model animals.

In this study, we established normal and heart failure pathological state models in rats and mice (heart failure with reduced ejection fraction [HFrEF] induced by anterior descending artery ligation in rats and heart failure with preserved ejection fraction [HFpEF] induced by “double hit” in mice). The objective was to investigate the differences in systolic/diastolic function changes between normal and failing hearts under different heart rate loads, as well as compare systolic/diastolic function between normal and failing hearts under the same heart rate load. The aim of this study was to improve the scientific rigor and standardization of experimental techniques by offering a foundation for regulating heart rate during echocardiographic tests in small animal models. This research enhances the scientific rigor and standardization of such studies by providing a foundation for heart rate control using echocardiography in small animal model experiments.

## 2 Materials and methods

### 2.1 Animals

Healthy male specific pathogen-free grade Sprague–Dawley rats (n=22; weight: 230–250 g) and healthy male specific pathogen-free grade C57BL/6N mice (n=22; weight: 20–24 g) aged 8 weeks were purchased from Beijing Vital River Laboratory Animal Technology Co., Ltd. (Beijing, China). The animals (3–5 per cage) were housed in barrier-level animal facilities at the Dongzhimen Hospital of Beijing University of Chinese Medicine (Beijing, China). They were maintained under conditions of constant temperature (20–26°C), constant humidity (60%), and a 12-h light-dark cycle, with free access to food and water. This study was approved by the Animal Ethics Committee of the Dongzhimen Hospital of Beijing University of Chinese Medicine (approval number: DZMYY24-02).

### 2.2 Study design

Rats were randomly classified into two groups using a computer-generated random number sequence: normal heart function and heart failure after myocardial infarction (MI). To ensure allocation concealment, the assignment sequence was kept in sequentially numbered, opaque, sealed envelopes by an independent researcher. A model was created by ligating the left anterior descending coronary artery. The exclusion criteria for the MI group included: (1) death during or within 24 hours post-surgery, (2) severe post-operative infection, and (3) failure to exhibit characteristic pathological Q waves on the electrocardiogram (ECG) on day 2 post-surgery. In this study, the mortality and failed model induction rate was 20-40%. The remaining successful model rats and normal rats were tracked using echocardiography on days 7 and 28 following surgery. Similarly, mice were also randomly assigned to a normal group and a “double hit” HFpEF model group using a random number table. After 12 weeks, the heart function of mice was tracked using echocardiography. To minimize operator bias, while complete blinding during the *in vivo* echocardiographic image acquisition was challenging due to the visible surgical scars in the MI models, all subsequent offline quantitative measurements and data analyses of the ultrasound images were performed by an independent investigator who was strictly blinded to the group allocations of both the rats and mice.

Induction anesthesia was performed using 5% isoflurane (600–700 ml/min in rats and 350–400 ml/min in mice), with the concentration adjusted during the ultrasound experiment to maintain anesthesia at 0.5–3.0%, resulting in a gradual decrease in heart rate. Rats had heart rates ranging 390–450, 320–380, and 240–300 bpm; while mice had heart rates ranging 450–550, 350–450, and 250–350 bpm. During these various ranges, heart rates, as well as cardiac contraction and relaxation functions were observed. Importantly, since heart rate regulation was achieved by titrating isoflurane concentrations, it must be explicitly acknowledged that the altered heart rate within this protocol was inherently inseparable from changes in the depth of anesthesia.

### 2.3 Establishment of rat model of heart failure after MI

The animals were weighed before receiving an intraperitoneal injection of 40 mg/kg of 1% sodium pentobarbital. After cleaning with alcohol, the left chest region was shaved. Tracheal intubation and preoperative ECG tests were carried out. Prior to opening the chest, the tracheal tube was attached to the small animal ventilator. Locate the left anterior descending coronary artery at the third left intercostal space. After making a cut along the intercostal gap (length: approximately 1.5 cm), the pleura, muscular layer, and subcutaneous tissue were separated layer by layer. The ribs were spread with a retractor so that the entire heart was visible. Using ophthalmic curved forceps (manufactured by Jinzhong Medical Instruments, Shanghai, China), the heart auricle was carefully lifted after tearing apart the pericardium. The origin of the left anterior descending branch of the coronary artery and its accompanying vein can be observed. Using the accompanying vein (the artery is on its right side) as a marker, a 5/0 sterile suture thread was inserted between the left atrial appendage and the pulmonary artery cone. With a depth of 2 mm and a width of 2–3 mm, the needle was inserted around 2–3 mm below the margin of the atrial appendage. Following ligation of the left anterior descending branch of the coronary artery and the vein that runs alongside it, the anterior chest wall of the rats appeared visibly pale. Using 2/0 sterile sutures, the skin, muscles, and chest cavity were closed, taking care to eliminate air from the chest cavity to avoid pneumothorax. Next, the ventilator was removed and the animal was assisted to begin breathing spontaneously. To prevent ventricular fibrillation, encourage diuresis, and lessen cardiac burden, 0.2 ml of lidocaine hydrochloride (manufactured by Otsuka Pharmaceutical (China) Co., Ltd.) and furosemide (manufactured by Tianjin Jin Yao Pharmaceutical Co., Ltd., Tianjin, China) were injected into the abdominal cavity of the animals immediately after surgery. To avoid infection, each animal received 400,000 IU of sodium penicillin (manufactured by North China Pharmaceutical Co., Ltd., Shijiazhuang, Hebei, China) intraperitoneally for 3 days consecutively following surgery.

ECG testing was used to assess the animals on day 2 following animal modeling. Briefly, 30 mg/kg of 1% sodium pentobarbital (imported and repackaged by Beijing Chemical Reagent Co., Ltd., Beijing, China) was injected intraperitoneally, and the 12-lead ECG of each animal was recorded. The model was deemed effective if 6–8 pathogenic Q waves appeared in the remaining leads, aside from leads II, III and AVF, according to the prior early evaluation and screening techniques established by the study team for heart failure models. An echocardiograph was used to track the heart function of rats on days 7 and 28 following surgery.

### 2.4 Establishment of “double hit” mouse HFpEF model

The mice in the model group received drinking water with a nitric oxide synthase inhibitor (L-NAME, 0.5 g/l, N5751; Sigma–Aldrich) to cause hypertension (pH = 7.4). In addition, high-fat diet with 60 kcal% fat (D12492; Research Diet) was administered to the mice to cause obesity and metabolic syndrome. Therefore, the HFpEF mouse model was produced following 12 weeks of feeding.

### 2.5 Image acquisition and analysis in echocardiography

The animals were placed in the ALC-ANE6 anesthesia machine (SHANGHAI ALCOTT BIOTECH Co., Ltd., Shanghai, China) and given a 5% isoflurane-oxygen mixture continuously for 1 min, or until the animal lost consciousness and the righting reflex turned negative. Position the animal’s mouth and nose under the anesthesia mask and continuously deliver isoflurane to maintain a state of light sedation. The isoflurane concentration is adjusted in real-time between 0.5–3.0% based on heart rate to ensure it remains within the range specified for each experimental condition.

To perform echocardiography, the Vevo2100 echocardiograph (manufactured by Visual Sonics Inc., Canada) with MS-250 and MS-400 probes, developed exclusively for rats and mice, was used. The probe center frequencies were 20 and 30 MHz, respectively. The coupling agent was preheated to prevent a low probe temperature that could cause sudden stimulation of the animal, thus leading to a decrease in body temperature and reduced cardiac function. The ultrasound machine was switched to two-dimensional mode and the probe was placed on the left ventricular long-axis portion adjacent to the sternum, ensuring that the direction of the probe matches the course of the left ventricle of the mouse. Subsequently, the high-frequency probe was raised to the plane of the left ventricular papillary muscle of the mouse and slowly rotated to 90° to obtain two-dimensional images of the left ventricular short-axis papillary muscle plane adjacent to the sternum. When the two-dimensional image showed the most distinct structure of the left ventricular papillary muscle, the M-mode sampling line was inserted vertically on the posterior wall of the left ventricle to obtain the M-mode ultrasound image of the left ventricle. The probe was positioned over the apex beat and the beam was aimed toward the right scapular region, while the mouse was positioned head-down on an inclined operation table. The mitral valve Doppler spectrum was obtained centrally within the left ventricular blood flow beam, and the pulsed Doppler sample volume was placed at the level of the mitral valve leaflets. Next, longitudinal motion velocities were measured by tissue Doppler examinations on the parietal and diaphragmatic sides of the mitral annulus. Each image must contain at least three consecutive cardiac cycles. The system simultaneously monitors the ECG, body temperature, respiration rate, and other fundamental metabolic parameters of the animal.

The following measurements were obtained by analyzing M-mode ultrasound images: left ventricular end-systolic anterior wall thickness, left ventricular end-diastolic anterior wall thickness, left ventricular end-systolic posterior wall thickness, left ventricular end-diastolic posterior wall thickness, left ventricular end-systolic internal diameter, left ventricular end-diastolic internal diameter, left ventricular ejection fraction (EF), and left ventricular short-axis shortening fraction (FS) and other systolic function indexes. Analysis of the Doppler ultrasound images of the mitral valve orifice is required to determine the peak early Doppler blood flow velocity (E) at the mitral valve, late diastolic peak Doppler blood flow velocity (A), isovolumetric relaxation time (IVRT), isovolumetric contraction time (IVCT), ejection time (ET), peak tissue Doppler velocity (e’) of myocardial relaxation at the mitral annulus during early diastole, and Tei index.

### 2.6 Statistical analysis

Statistical analysis was performed using SPSS Statistics 22.0 software (IBM Corp., Armonk, NY, USA). Quantitative data with a normal distribution were described as mean ± standard deviation. An independent samples t-test was used to compare two groups, because echocardiographic parameters across the three heart rate ranges were sequentially measured within the same individual animals, a repeated measures analysis of variance (RM-ANOVA) was utilized to evaluate these intra-group differences, followed by Bonferroni’s *post hoc* test for pairwise comparisons. Non-normally distributed quantitative data were described using the median and interquartile range (25th, 75th percentile). Nonparametric tests were used for intergroup comparisons. The Mann–Whitney U and Kruskal–Wallis H tests were used for two- and multiple-group comparisons, respectively. Simple linear regression analysis was applied to evaluate the relationship between heart rate and cardiac function parameters that exhibited linear trends. The confidence interval was established at 95% for all cases, and p-values <0.05 denote statistically significant difference.

## 3 Results

### 3.1 Anesthetic conditions

Induction anesthesia was performed using isoflurane at a constant concentration of 5%. The flow rate was changed to varying degrees to maintain the heart rate of anaesthetized animals close to that of awake animals after induction anesthesia. The model group required a lower induction anesthesia flow rate than the normal group. The concentration and flow rate required to maintain anesthesia were then adjusted using a closed nasal cannula gradient reduction. The effects of various heart rate ranges on cardiac function in normal and model animals were investigated (**Table 1**).

**Table 1:** Anaesthetic conditions.

Group (n=11)	Body weight (g)	Induction anaesthesia (concentration, flow rate)	Maintain anaesthesia (concentration, flow rate)					
			500 ± 50bpm	420 ± 30bpm	400 ± 50bpm	350 ± 30bpm	300 ± 50bpm	270 ± 30bpm
NC-R	275.18 ± 13.38	5%, 700ml /min	–	1.5~2.0%, 260~270 ml /min	–	2.0~2.5%, 180~200 ml /min	–	2.8~3.0%, 180~200ml /min
MI7D-R	358.09 ± 16.34	5%, 600ml /min	–	1.5~2.0%, 260~270 ml /min	–	2.0~2.5%, 180~200 ml /min	–	2.8~3.0%, 180~200ml /min
MI28D-R	398.82 ± 30.20	5%, 600ml /min	–	1.5~2.0%, 260~270 ml /min	–	2.0~2.5%, 180~200 ml /min	–	2.8~3.0%, 180~200ml /min
NC-M	24.00 ± 0.92	5%, 400ml /min	0.6~1.0%, 80~100ml /min	–	1.0~1.5%, 80~100ml /min	–	2.0~3.0%, 80~100ml /min	–
HFpEF-M	45.55 ± 3.01	5%, 350ml /min	0.6~1.0%, 80~100ml /min	–	1.0~1.5%, 80~100ml /min	–	2.0~3.0%, 80~100ml /min	–

bpm, Beat per minute; HFpEF-M, Heart failure with preserved ejection fraction mice; MI7D-R, Rats in the 7-day post-myocardial infarction group; MI28D-R, Rats in the 28-day post-myocardial infarction group; NC-R, Normal control goup rats; NC-M, Normal control goup mice.

### 3.2 Model evaluation

**Figure 1:**
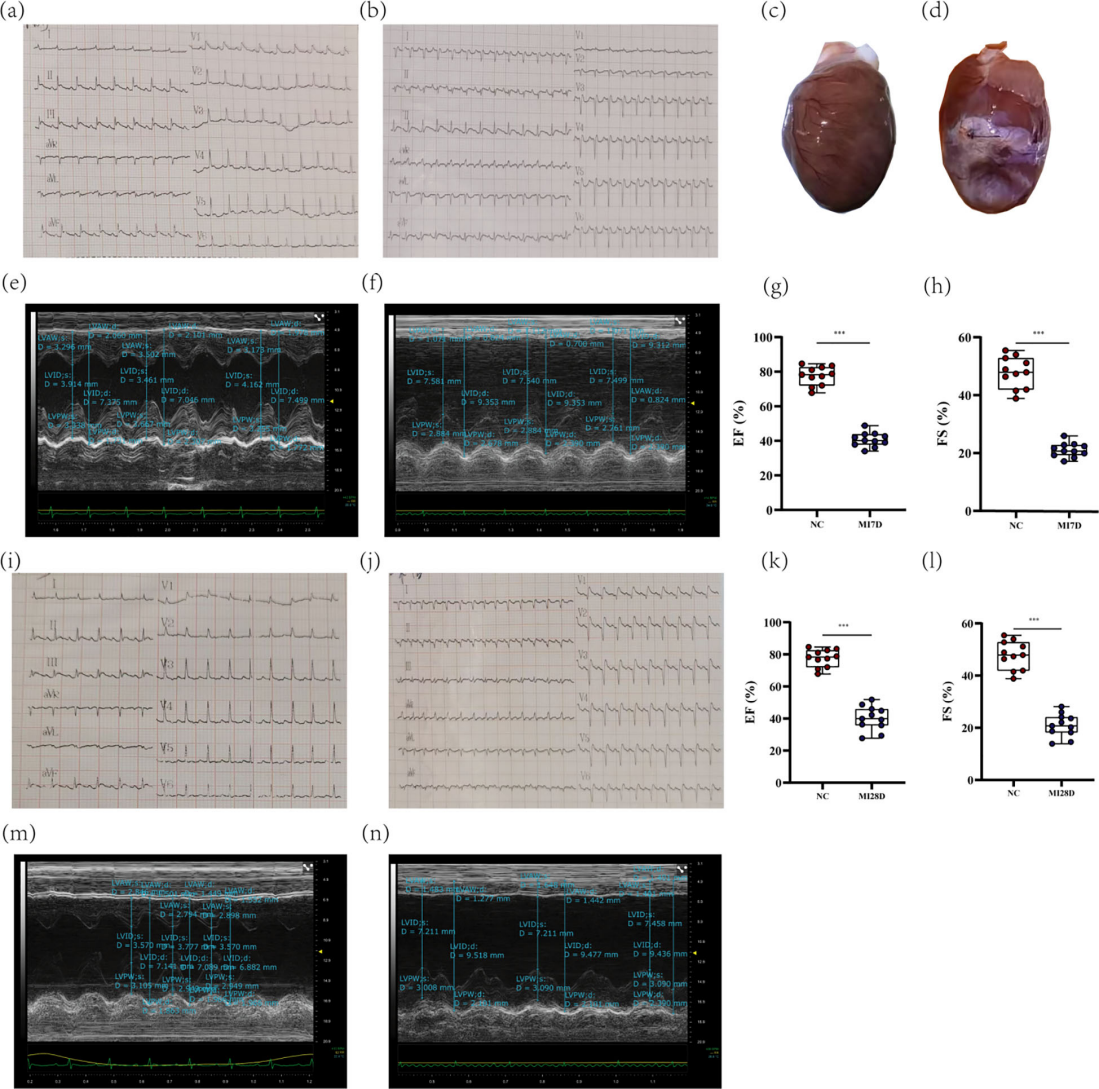
Evaluation of MI7D and MI28D rats. **(a, i)** Electrocardiogram of normal control rats; **(b)** Electrocardiogram of rats 7 days after myocardial infarction-induced heart failure; **(c)** Heart image of normal control rats; **(d)** Heart image of rats 7 days after myocardial infarction-induced heart failure; **(e, m)** Echocardiogram of normal control rats; **(f)** Echocardiography of rats 7 days after myocardial infarction with heart failure; **(g, h)** LVEF and FS values between the normal control group and the group 7 days after myocardial infarction with heart failure; **(j)** Electrocardiogram of rats 28 days after myocardial infarction-induced heart failure; **(k, l)** LVEF and FS values between the normal control group and the group 28 days after myocardial infarction with heart failure; **(n)** Echocardiography of rats 28 days after myocardial infarction with heart failure. *** indicates P < 0.001 vs. Normal Control group.

As illustrated in **Figure 1**, rats in the normal group had normal ECG prior to surgery. On day 2 after surgery, rats in the model group displayed clear pathological Q waves, which were primarily focused in leads V3-6, I, and AVL, with the decrease of EF value. Under ultrasound, rats in the normal group had good ventricular wall motion, displaying M-type waves with regular motion. On days 7 and 28, anterior wall motion was weakened or even eliminated in rats with heart failure in the model group.

**Figure 2:**
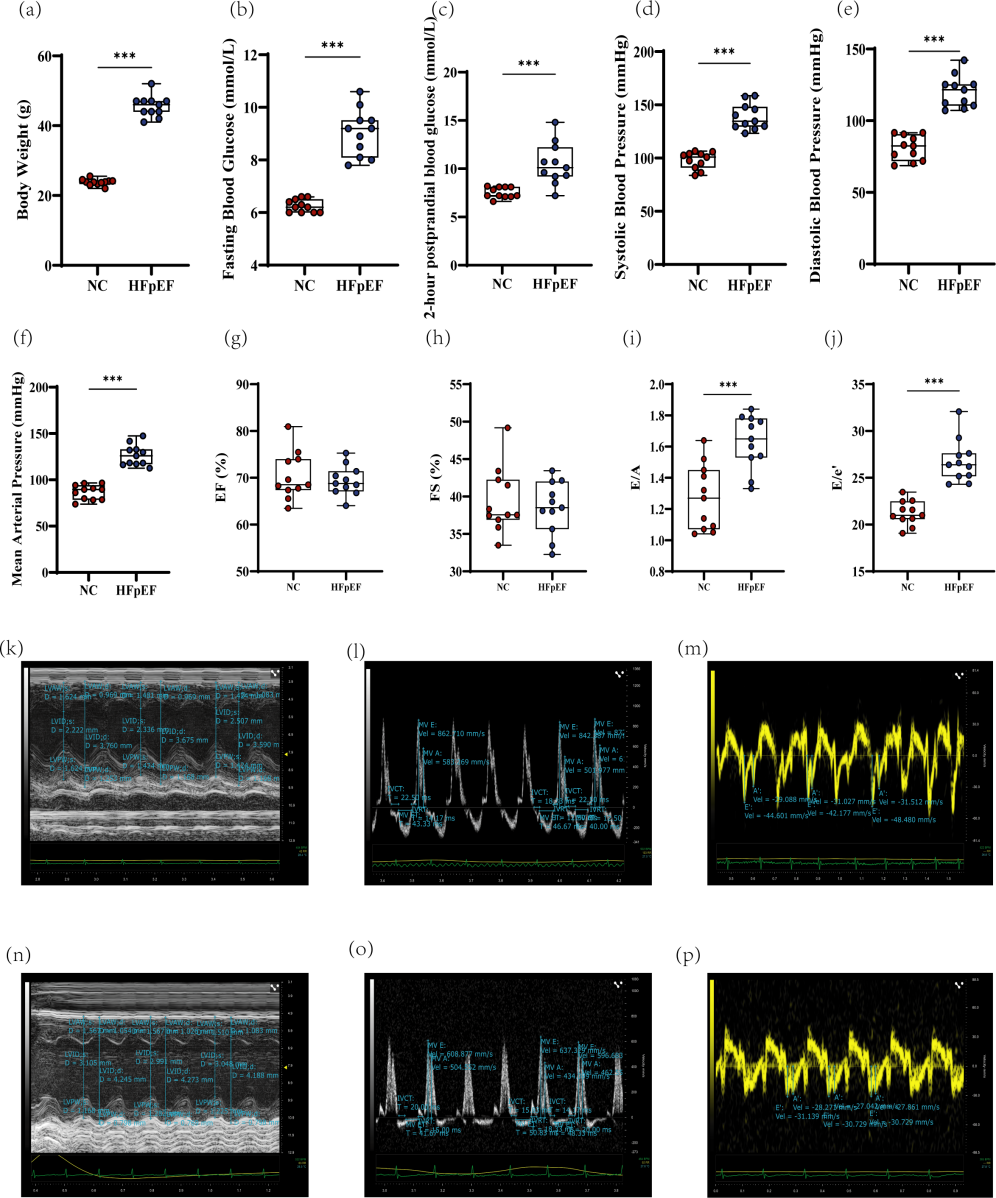
Model evaluation mice. **(a)** Body weight between normal control and HFpEF mice; **(b)** Fasting blood glucose between normal control and HFpEF mice; **(c)** 2-hour postprandial blood glucose between normal control and HFpEF mice; **(d)** Systolic blood pressure between normal control and HFpEF mice; **(e)** Diastolic blood pressure between normal control and HFpEF mice; **(f)** Mean arterial pressure between normal control and HFpEF mice; **(g)** LVEF values between normal control and HFpEF mice; **(h)** FS values between normal control and HFpEF mice; **(i)** E/A ratios between normal control and HFpEF mice; **(j)** E/e’ values between normal control and HFpEF mice; **(k–m)** Echocardiograms of normal control mice; **(n–p)** Echocardiograms of HFpEF mice. *** indicates P < 0.001 vs. Normal Control group.

As demonstrated in **Figure 2**, 12 weeks of high-fat diet with L-NAME treatment resulted in obesity, hypertension, and decreased glucose tolerance in mice. In cardiac ultrasound, we found diastolic dysfunction but no change in systolic EF.

### 3.3 Differences in rat heart function measured by ultrasound at different heart rates

**Figure 3:**
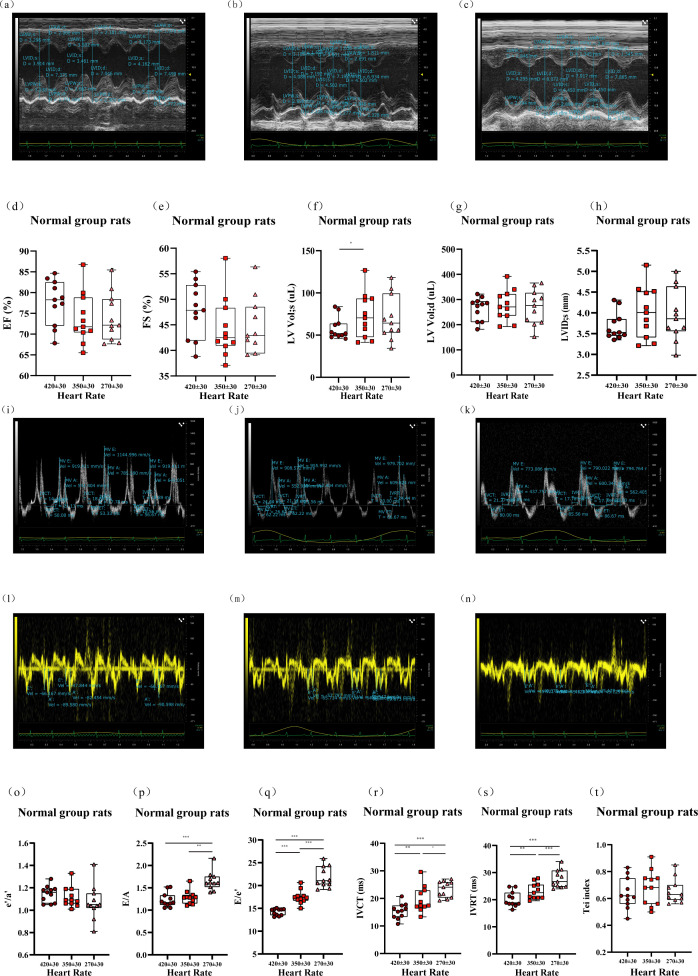
Cardiac function in normal rats at different heart rates. **(a–c)** Echocardiograms of normal rats at heart rates of 420 ± 30 bpm, 350 ± 30 bpm, and 270 ± 30 bpm; **(d)** LVEF values in normal rats across three heart rate ranges; **(e)** FS values in normal rats across three heart rate ranges; **(f)** LV Vols values in the normal group at three heart rate ranges; **(g)** LV Vold values in the normal group at three heart rate ranges; **(h)** LVIDs values in the normal group at three heart rate ranges; **(i–n)** Echocardiographic images of diastolic function in the normal group at heart rates of 420 ± 30 bpm, 350 ± 30 bpm, and 270 ± 30 bpm; **(o)** e’/a’ values in normal group rats across three heart rate ranges; **(p)** E/A values in normal group rats across three heart rate ranges; **(q)** E/e’ values in normal rats across three heart rate ranges; **(r)** IVCT values in normal rats across three heart rate ranges; **(s)** IVRT values in normal rats across three heart rate ranges; **(t)** Tei indices in normal rats across three heart rate ranges. * indicates P < 0.05, ** indicates P < 0.01, and *** indicates P < 0.001.

**Figure 4:**
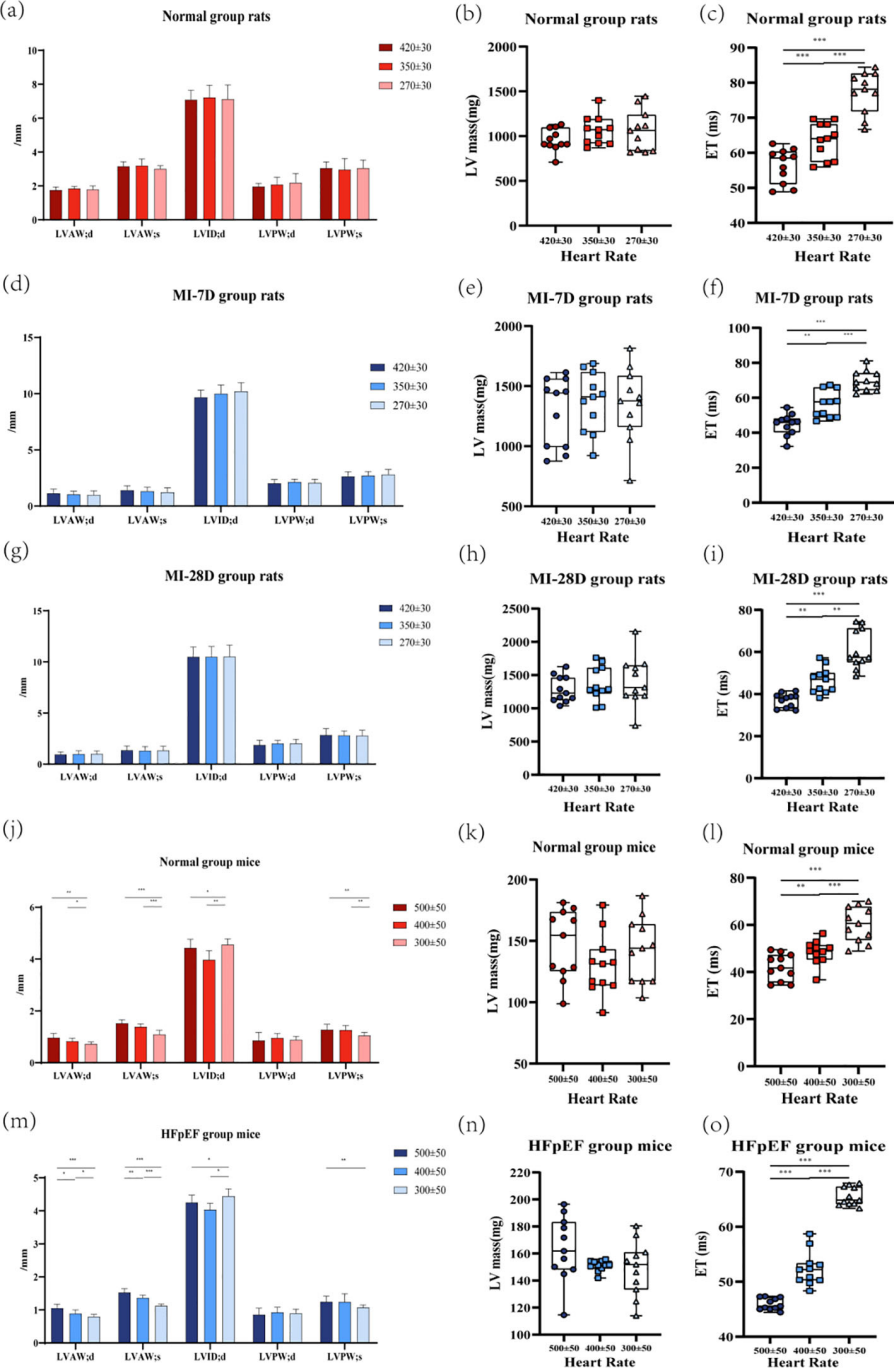
Other cardiac function parameters. **(a–c)** Changes in indicators of systolic and diastolic function in normal rats at different heart rates; **(d–f)** Indicators of systolic and diastolic function in rats in the MI7D model group; **(g–i)** Indicators of systolic and diastolic function in rats in the MI28D model group; **(j–l)** Changes in indicators related to systolic and diastolic function in the normal group of mice at different heart rates; **(m–o)** Indicators related to systolic and diastolic function in the HFpEF model group of mice. * indicates P < 0.05, ** indicates P < 0.01, and *** indicates P < 0.001.

Firstly, we used ultrasound to test the cardiac function of rats in the normal group at different heart rates. **Figure 3** shows that as the heart rate range decreased, the absolute values of E/e’ and IVRT, which are systolic function parameters, were markedly increased. A decrease in heart rate to 270 ± 30 bpm resulted in a significant increase in the E/A ratio compared with the 420 ± 30 and 350 ± 30 bpm group. This finding suggests that a low heart rate can negatively impact normal diastolic function. Compared with the 420 ± 30 bpm group, the left ventricular systolic volume in 350 ± 30 bpm group was significantly increased. Progressive reductions in heart rate were accompanied by marked increases in the IVCT and ET. Nonetheless, there was no obvious statistically significant difference in other systolic function, such as EF and FS, seen **Figure 4**.

**Figure 5:**
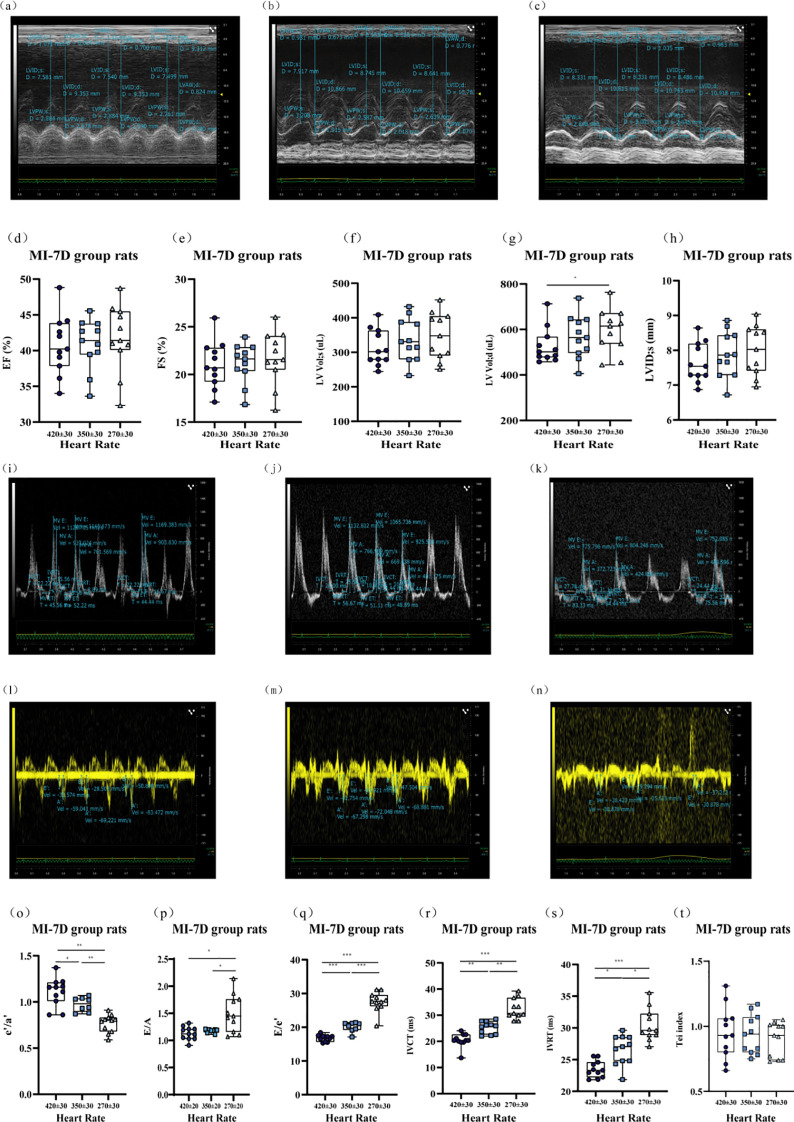
Cardiac function in MI7D rats at different heart rates. **(a–c)** Echocardiographic images of systolic function in MI7D rats at heart rates of 420 ± 30 bpm, 350 ± 30 bpm, and 270 ± 30 bpm, respectively; **(d)** LVEF values in MI7D rats across three heart rate ranges; **(e)** FS values in MI7D rats across three heart rate ranges; **(f)** LV Vols values in MI7D rats across three heart rate ranges; **(g)** LV Vold values in MI7D rats across three heart rate ranges; **(h)** LVIDs values in MI7D rats across three heart rate ranges; **(i-n)** Echocardiographic images of diastolic function in MI7D rats at heart rates of 420 ± 30 bpm, 350 ± 30 bpm, and 270 ± 30 bpm; **(o)** e’/a’ values in MI7D rats across three heart rate ranges; **(p)** E/A values in MI7D rats across three heart rate ranges; **(q)** E/e’ values in MI7D rats across three heart rate ranges; **(r)** IVCT values in MI7D rats across three heart rate ranges; **(s)** IVRT values in MI7D rats across three heart rate ranges; **(t)** Tei index in MI7D rats across three heart rate ranges. * indicates P < 0.05, ** indicates P < 0.01, and *** indicates P < 0.001.

Secondly, we performed echocardiography measurements in rats from the model group of heart failure 7 and 28 days after MI modelling. Seven days after MI, rats with heart failure exhibited a decrease in heart rate from 420 ± 30 to 350 ± 30 and 270 ± 30 bpm. The E/A ratio was significantly higher at a heart rate of 270 ± 30 bpm than at 420 ± 30 or 350 ± 30 bpm. A gradient decrease in heart rate resulted in marked elevations in the absolute values of E/e’, e’/a’ and IVRT. These findings indicated abnormal diastolic dysfunction at low heart rates. The diastolic volumes of the left ventricle as well showed a significant increase in the group with a heart rate range of 270 ± 30 bpm compared with the 420 ± 30 bpm group. Significant increases in the IVCT and ET coincided with progressive heart rate reductions. No significant changes were observed in other systolic function parameters, such as EF and FS values. (**Figures 4**, **5**).

**Figure 6:**
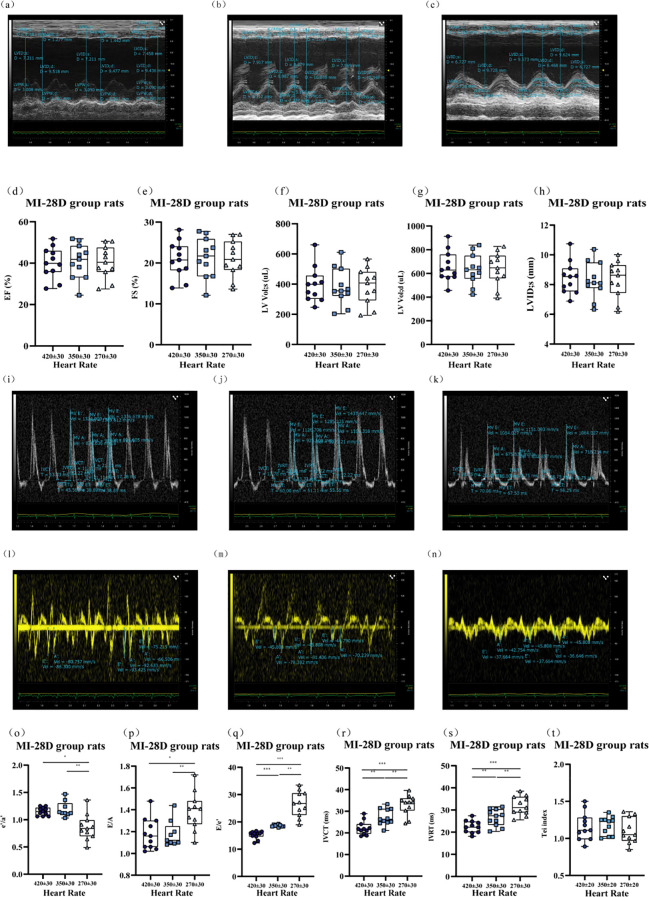
Cardiac function of MI28D rats at different heart rates. **(a–c)** Echocardiographic images of systolic function in MI28D rats at heart rates of 420 ± 30 bpm, 350 ± 30 bpm, and 270 ± 30 bpm, respectively; **(d)** LVEF values in MI28D rats across three heart rate ranges; **(e)** FS values in MI28D rats across three heart rate ranges; **(f)** LV Vols values in MI28D rats across three heart rate ranges; **(g)** LV Vold values in MI28D rats across three heart rate ranges; **(h)** LVIDs values in MI28D rats across three heart rate ranges; **(i-n)** Echocardiographic assessment of diastolic function in MI28D rats at heart rates of 420 ± 30 bpm, 350 ± 30 bpm, and 270 ± 30 bpm; **(o)** e’/a’ values in MI28D rats across three heart rate ranges; **(p)** E/A values in MI28D rats across three heart rate ranges; **(q)** E/e’ values in MI28D rats across three heart rate ranges; **(r)** IVCT values in MI28D rats across three heart rate ranges; **(s)** IVRT values in MI28D rats across three heart rate ranges; **(t)** Tei index in MI28D rats across three heart rate ranges. * indicates P < 0.05, ** indicates P < 0.01, and *** indicates P < 0.001.

As shown in **Figure 6**, compared with the group with a heart rate range of 420 ± 30 and 350 ± 30 bpm, the E/A ratio were significantly increased in rats with MI at 28 days in the group with a heart rate range of 270 ± 30 bpm. The e’/a’ ratio was significantly lower at a heart rate of 270 ± 30 bpm than at 420 ± 30 or 350 ± 30 bpm. Furthermore, progressive reductions in heart rate were accompanied by marked increases in the E/e’ and IVRT. The same results can also be observed in the contractile function indices IVCT and ET. Other systolic function parameters, including EF and FS, did not exhibit significant alterations. Additional data on echocardiographic parameters of systolic and diastolic function in rats with MI at 28 days can be found in the appendix **Figure 4**.

**Figure 7:**
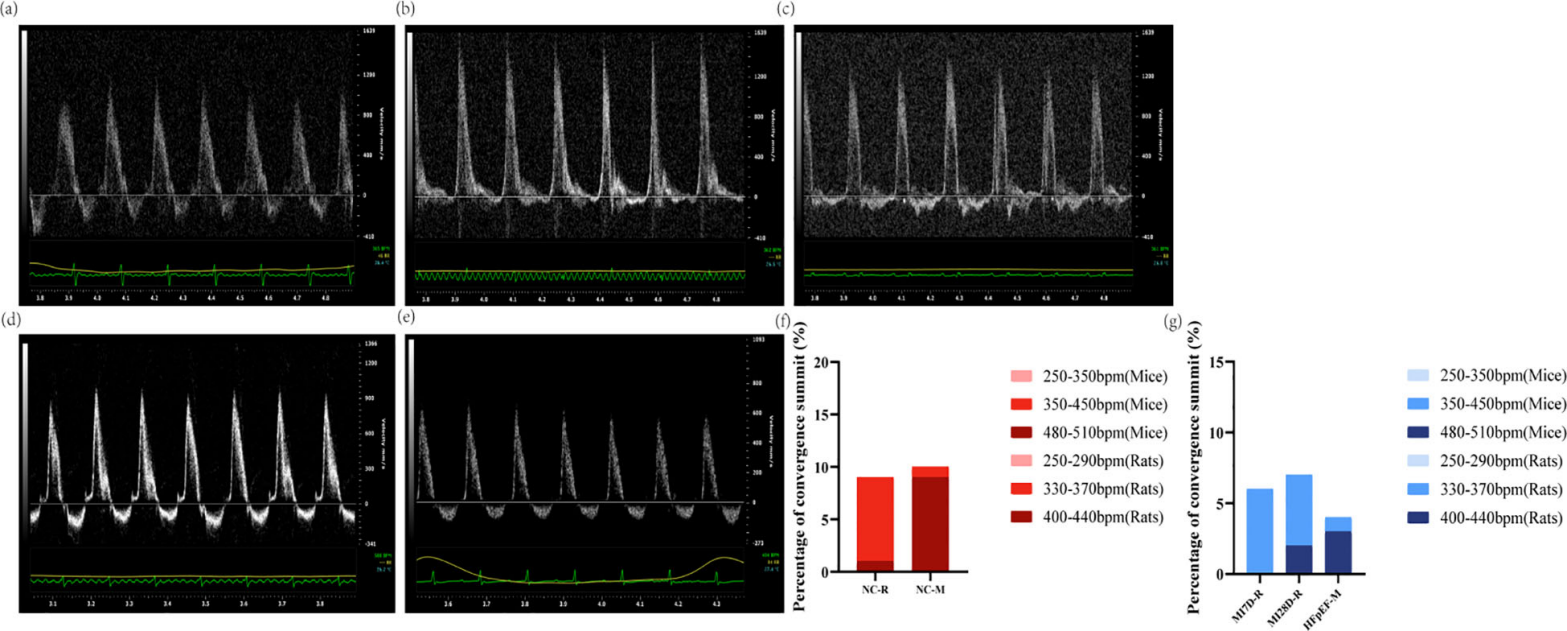
Fusion peaks. **(a–c)** Fusion peaks in rats at heart rates of 330–370 bpm; **(d, e)** Fusion peaks in mice at heart rates of 480–510 bpm; **(f)** Occurrence and proportion of fusion peaks in normal rats and mice; **(g)** Occurrence and proportion of fusion peaks in model rats and mice. * indicates P < 0.05, ** indicates P < 0.01, and *** indicates P < 0.001.

Interestingly, we observed that when the heart rate ranged 320–380 bpm, isolated instances of E/A fusion peaks occurred in the normal group and in rats with heart failure on days 7 and 28 post-MI. This phenomenon prevented the measurement of E wave, A wave, and e’ wave parameters, with incidence rates of 72.73% (8/11), 54.55% (6/11), and 45.45% (5/11), respectively. At heart rates ranging 400–440 bpm, the incidence of fused peaks decreased across all three groups to 9.09% (1/11), 0% (0/11), and 18.18% (2/11), respectively. No fused peaks were observed at heart rates ranging 250–290 bpm. Normal and HFpEF model mice exhibited a high incidence of fused peaks at heart rates of 480–510 bpm, with rates of 81.82% (9/11) and 27.27% (3/11), respectively. When the heart rate decreased to 350–450 bpm, the incidence of fused peaks similarly declined to 9.09% (1/11) and 9.09% (1/11), respectively. No fused peaks were observed at heart rates ranging 250–290 bpm (**Figure 7**).

### 3.4 Differences in cardiac function measured by ultrasound in normal and HFpEF mice at different heart rates

**Figure 8:**
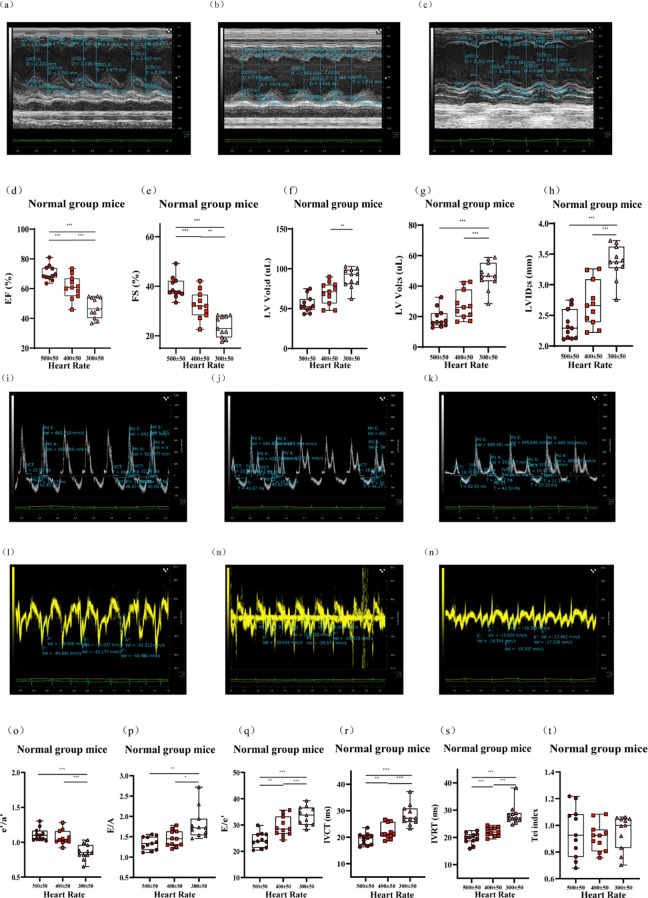
Cardiac function in normal mice at different heart rates. **(a–c)** Echocardiographic images of systolic function in normal mice at heart rates of 420 ± 30 bpm, 350 ± 30 bpm, and 270 ± 30 bpm, respectively; **(d)** LVEF values in normal group mice across three heart rate ranges; **(e)** FS values in normal group mice across three heart rate ranges; **(f)** LV Vols values in the normal group at three heart rate ranges; **(g)** LV Vold values in the normal group at three heart rate ranges; **(h)** LVIDs values in the normal group at three heart rate ranges; **(i–n)** Echocardiographic images of diastolic function in the normal group at heart rates of 420 ± 30 bpm, 350 ± 30 bpm, and 270 ± 30 bpm; **(o)** e’/a’ values in normal group mice across three heart rate ranges; **(p)** E/A values in normal group mice across three heart rate ranges; **(q)** E/e’ values in normal group mice across three heart rate ranges; **(r)** IVCT values in normal group mice across three heart rate ranges; **(s)** IVRT values in normal group mice across three heart rate ranges; **(t)** Tei index in normal group mice across three heart rate ranges. * indicates P < 0.05, ** indicates P < 0.01, and *** indicates P < 0.001.

**Figure 8** shows that as the heart rate gradient decreases, both E/e’ and IVRT progressively increase, with statistically significant differences observed between each heart rate interval. Compared with the group with a heart rate range of 500 ± 50 and 400 ± 50 bpm, the E/A ratio were significantly increased in the group with a heart rate range of 300 ± 50 bpm. The e’/a’ ratio was significantly lower at a heart rate of 300 ± 50 bpm than at 500 ± 50 or 400 ± 50 bpm. Systolic function (EF, FS) declines markedly with slowing heart rate, while IVCT and ET show absolute prolongation. Compared with the group of 500 ± 50 bpm and 400 ± 50 bpm, the 300 ± 50 bpm group showed a significant increase in both left ventricular systolic volume and left ventricular systolic internal diameter. The left ventricular diastolic volume was significantly higher in the 300 ± 50 bpm heart rate group than in the 400 ± 50 bpm heart rate group. Other systolic function parameters in normal mice can be found in the appendix **Figure 4**.

**Figure 9:**
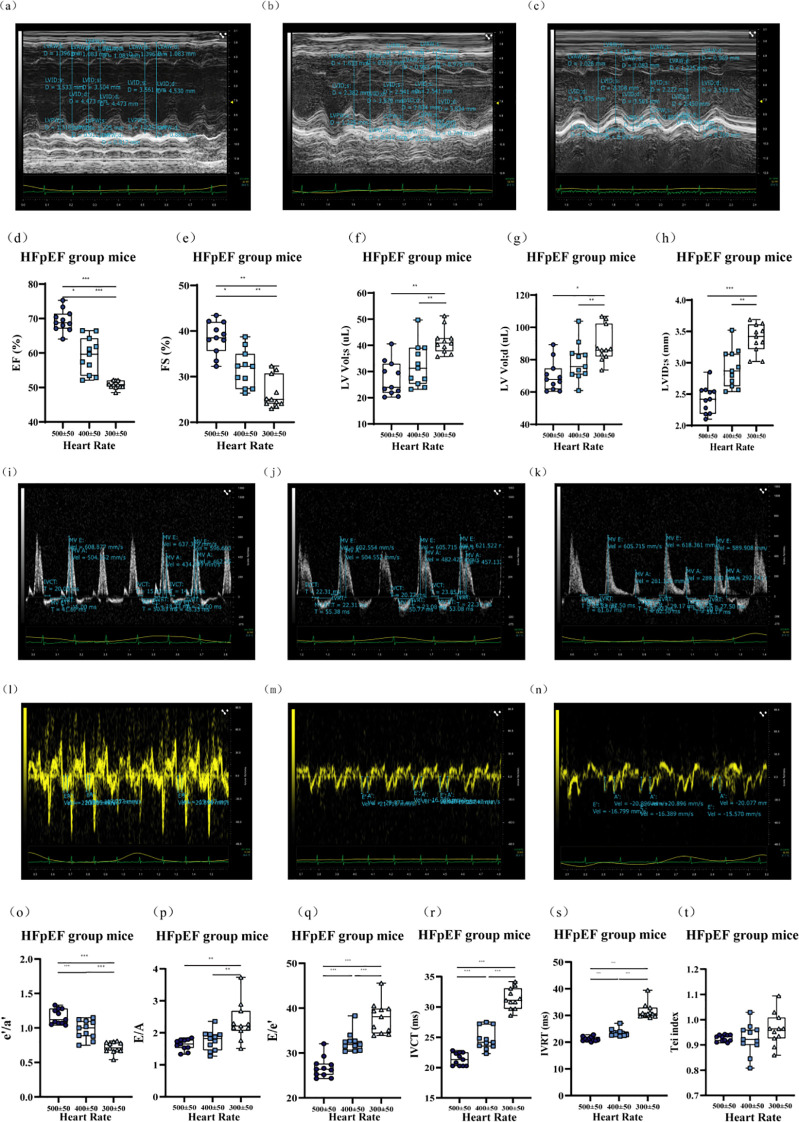
Cardiac function in HFpEF mice at different heart rates. **(a–c)** Echocardiographic images of systolic function in HFpEF mice at heart rates of 420 ± 30 bpm, 350 ± 30 bpm, and 270 ± 30 bpm, respectively; **(d)** LVEF values in HFpEF mice across three heart rate ranges; **(e)** FS values in HFpEF mice across three heart rate ranges; **(f)** LV Vols values in HFpEF mice across three heart rate ranges at three heart rate ranges; **(g)** LV Vold in HFpEF mice at three heart rate ranges; **(h)** LVIDs in HFpEF mice at three heart rate ranges; **(i–n)** Echocardiographic assessment of diastolic function in HFpEF mice at heart rates of 420 ± 30 bpm, 350 ± 30 bpm, and 270 ± 30 bpm; **(o)** e’/a’ values in HFpEF mice across three heart rate ranges; **(p)** E/A values in HFpEF mice across three heart rate ranges; **(q)** E/e’ values in HFpEF mice across three heart rate ranges; **(r)** IVCT values in HFpEF mice across three heart rate ranges; **(s)** IVRT values in HFpEF mice across three heart rate ranges; **(t)** Tei index in HFpEF mice across three heart rate ranges. * indicates P < 0.05, ** indicates P < 0.01, and *** indicates P < 0.001.

In **Figure 9**, as heart rate decreased, both E/e’, e’/a’ and IVRT showed a significant increase in comparisons between each heart rate range in the HFpEF mouse model. Compared with the 500 ± 50 and 400 ± 50 ranges, E/A showed a significant increase in the 300 ± 50 bpm group, which exhibited marked impairment in diastolic function parameters. Simultaneously, systolic function parameters (EF, FS, LVIDs, etc.) were significantly affected, seen **Figure 4** in appendix.

### 3.5 Evaluation of cardiac function difference between normal and heart failure rats by ultrasound under the same heart rate

**Figure 10:**
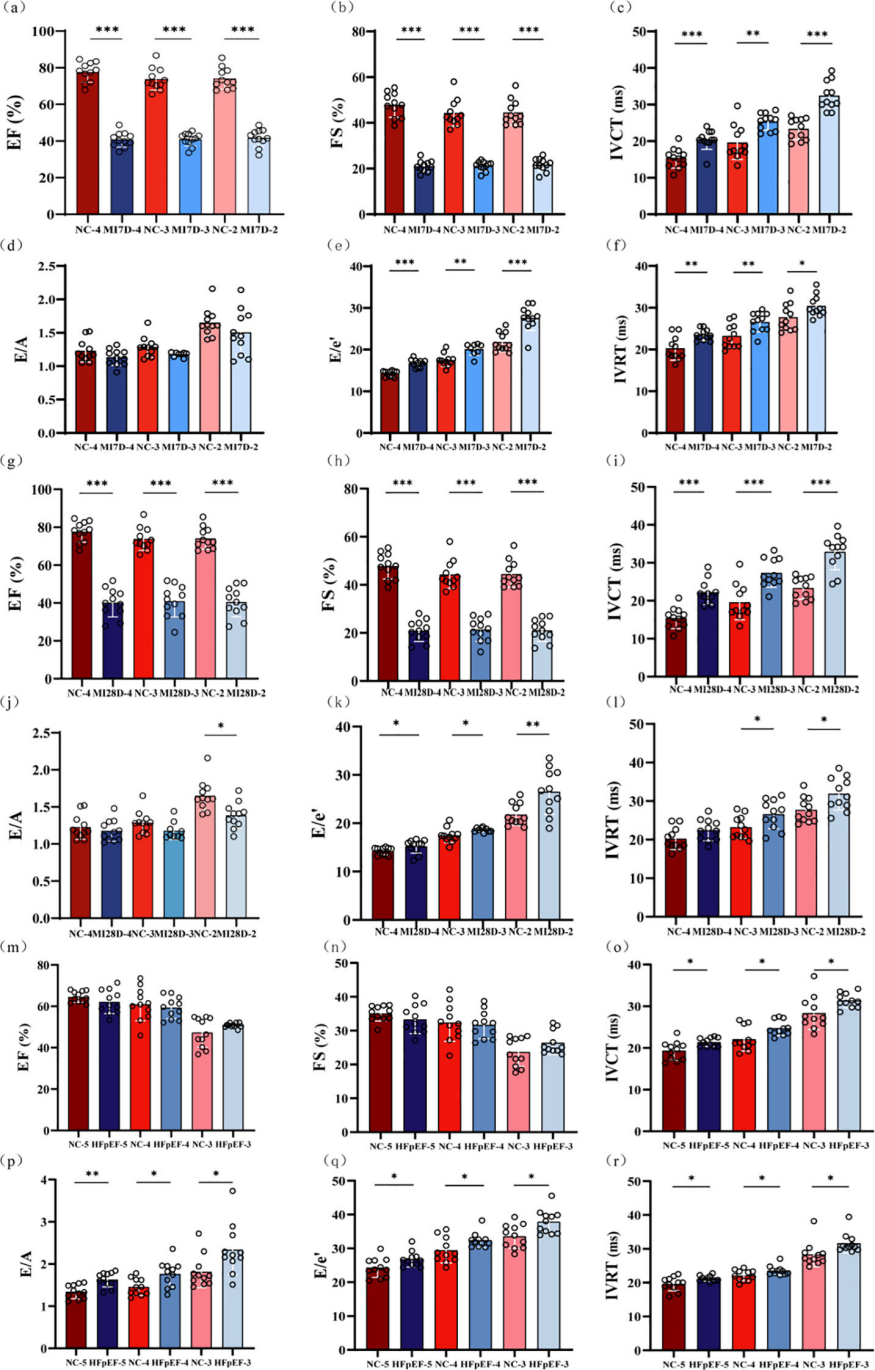
Cardiac function in normal rats/mice versus model rats/mice at the same heart rate. **(a)** LVEF values between normal and MI7D rats; **(b)** FS between normal and MI7D rats; **(c)** IVCT values between normal and MI7D rats; **(d)** E/A values between normal and MI7D rats; **(e)** E/e’ values between normal and MI7D rats; **(f)** IVRT values between normal and MI7D rats; **(g)** LVEF values between normal and MI28D rats; **(h)** FS between normal and MI28D rats; **(i)** IVCT values between normal and MI28D rats; **(j)** E/A values between normal and MI28D rats; **(k)** E/e’ values between normal and MI28D rats; **(l)** IVRT values between normal and MI28D rats; **(m)** LVEF values between normal and HFpEF mice; **(n)** FS between normal and HFpEF mice; **(o)** IVCT values between normal and HFpEF mice; **(p)** E/A values between normal and HFpEF mice; **(q)** E/e’ values between normal and HFpEF mice; **(r)** IVRT values between normal and HFpEF mice. * indicates P < 0.05, ** indicates P < 0.01, and *** indicates P < 0.001.

When comparing heart rates within the range of 420 ± 30 bpm, rats with heart failure 7 days post-MI exhibited significantly reduced systolic function (EF, FS) and markedly prolonged absolute IVCT compared with those in the normal group (**Figures 10a–f**). Dilation parameters (E/e’, IVRT) were also significantly increased. When comparing heart rates within the ranges of 350 ± 30 and 270 ± 30 bpm, rats with heart failure 7 days post-MI exhibited significantly reduced systolic function (EF, FS) and markedly prolonged absolute IVCT compared with those in the normal group. Deterioration in diastolic function (E/e’, IVRT) was also significantly impaired. When comparing heart rates within the range of 420 ± 30, rats with heart failure 28 days post-MI exhibited significantly reduced systolic function (LVEF, FS) compared with those in the normal group, along with prolonged absolute IVCT values and markedly abnormal diastolic function (E/e’) (**Figures 10g–l**). IVRT showed an upward trend in the model group. At heart rates ranging 350 ± 30 bpm, rats with heart failure 28 days after MI showed significantly decreased systolic function (EF, FS), prolonged absolute IVCT, and markedly impaired diastolic function (E/e’, IVRT) compared with those in the normal group. At heart rates ranging 270 ± 30 bpm, rats with heart failure 28 days after MI exhibited significantly reduced systolic function (EF, FS), prolonged absolute IVCT, and markedly impaired diastolic function (E/A, E/e’, IVRT) compared with those in the normal group.

### 3.6 Comparison of ultrasonic evaluation of cardiac function between normal and HFpEF mice under the same heart rate

When the heart rates ranged 500 ± 50 bpm, EF and FS showed no changes. However, in the model group, the absolute values of IVCT were prolonged, and E/A, E/e’, and IVRT were significantly elevated. These results suggested that mice in the HFpEF group had preserved EF but impaired diastolic function. At a heart rate range of 400 ± 50 bpm, E/A, E/e’, IVCT, and IVRT were significantly elevated in the model group, while EF and FS remained unchanged. At heart rates ranging 300 ± 50 bpm, no statistically significant differences were observed in EF or FS. The absolute IVCT value was prolonged, and common parameters of diastolic function—E/A, E/e’, and IVRT—were significantly elevated in the model group, accompanied by a marked decrease in e’/a’ (**Figures 10m–r**).

### 3.7 Relationship between heart rate and cardiac function

The above results demonstrated that cardiac function fluctuates significantly with heart rate. Therefore, we performed simple linear regression analysis on commonly used diastolic function indicators (E/A, E/e’, IVRT) and systolic function indicators (EF, FS, IVCT, ET) between normal and model group rats to further analyze their linear dependence.

**Figure 11:**
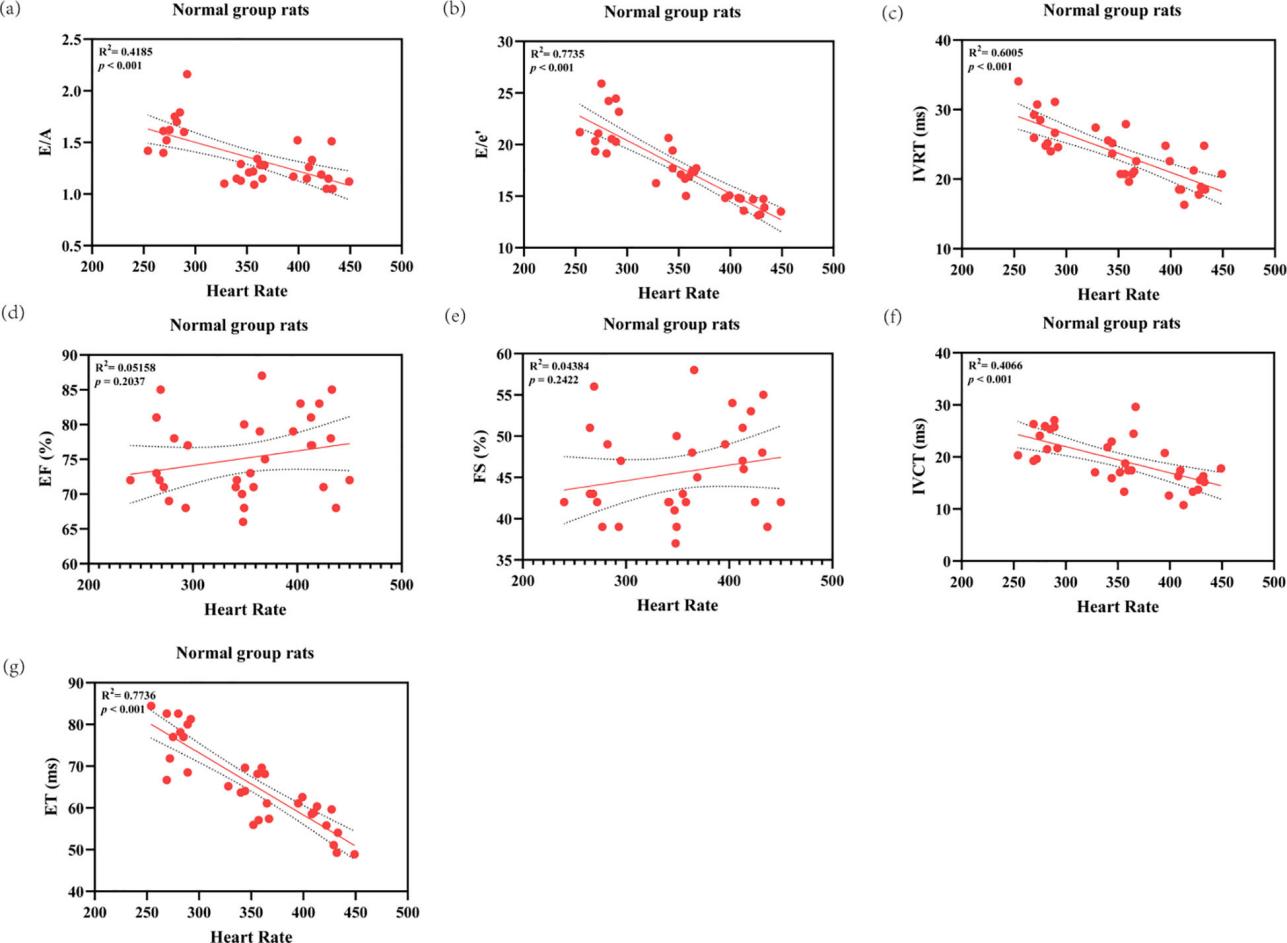
Correlation analysis in normal rats. **(a)** Correlation analysis of E/A ratio in normal rats; **(b)** Correlation analysis of E/e’ ratio in normal rats; **(c)** Correlation analysis of IVRT in normal rats; **(d)** Correlation analysis of LVEF values in normal rats; **(e)** Correlation analysis of FS values in normal rats; **(f)** Correlation analysis of IVCT in normal rats; **(g)** Correlation analysis of ET in normal rats.

**Figure 12:**
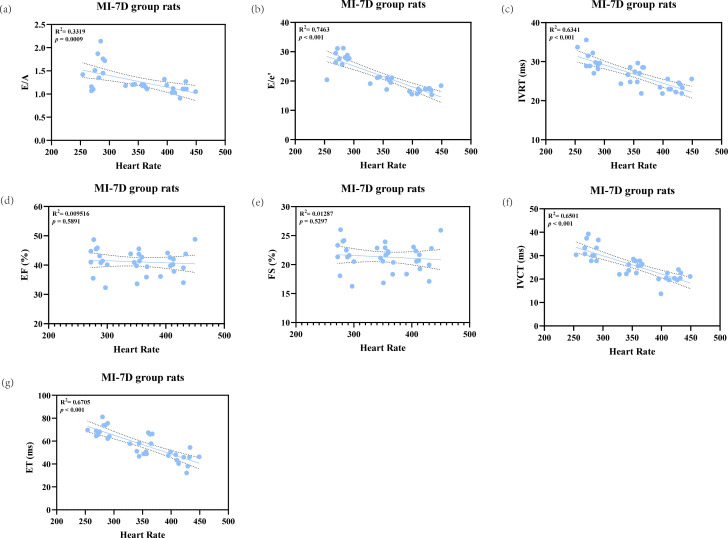
Correlation analysis in normal MI7D rats. **(a)** Correlation analysis of E/A ratio in MI7D rats; **(b)** Correlation analysis of E/e’ ratio in MI7D rats; **(c)** Correlation analysis of IVRT in MI7D rats; **(d)** Correlation analysis of LVEF values in MI7D rats; **(e)** Correlation analysis of FS values in MI7D rats; **(f)** Correlation analysis of IVCT in MI7D rats; **(g)** Correlation analysis of ET in MI7D rats.

**Figure 13:**
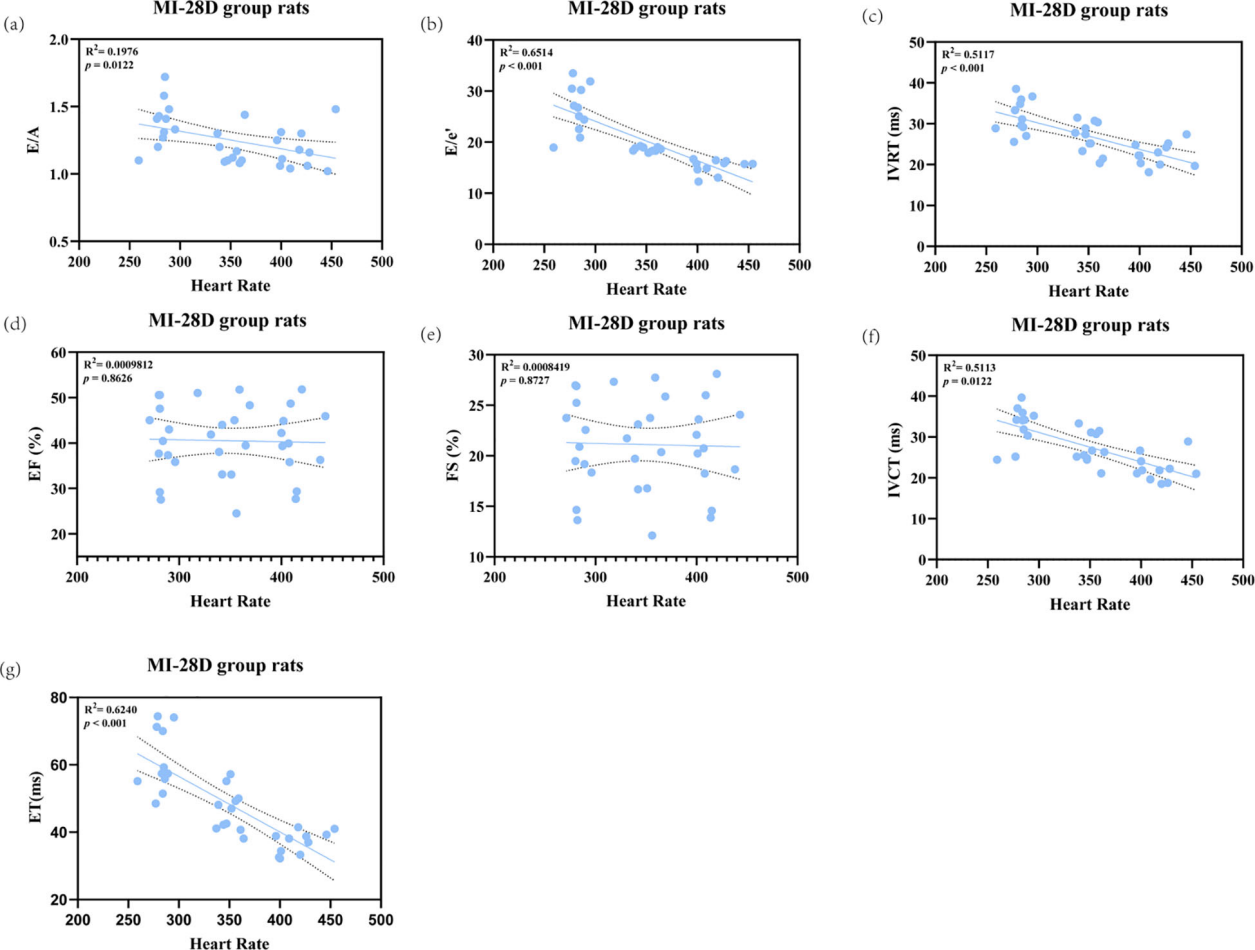
Correlation analysis in normal MI28D rats. **(a)** Correlation analysis of E/A ratio in MI28D rats; **(b)** Correlation analysis of E/e’ ratio in MI28D rats; **(c)** Correlation analysis of IVRT in MI28D rats; **(d)** Correlation analysis of LVEF values in MI28D rats; **(e)** Correlation analysis of FS values in MI28D rats; **(f)** Correlation analysis of IVCT in MI28D rats; **(g)** Correlation analysis of ET in MI28D rats.

**Figure 14:**
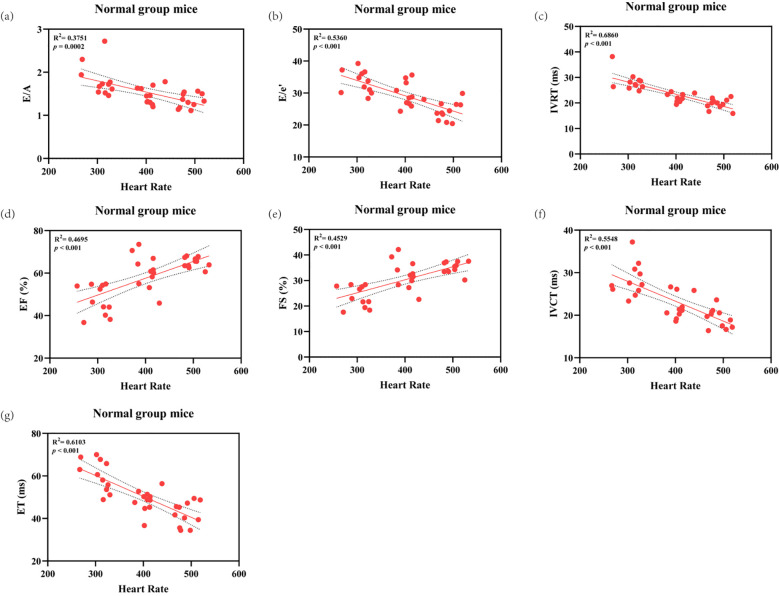
Correlation analysis in normal mice. **(a)** Correlation analysis of E/A ratio in normal mice; **(b)** Correlation analysis of E/e’ ratio in normal mice; **(c)** Correlation analysis of IVRT in normal mice; **(d)** Correlation analysis of LVEF values in normal mice; **(e)** Correlation analysis of FS values in normal mice; **(f)** Correlation analysis of IVCT in normal mice; **(g)** Correlation analysis of ET in normal mice.

**Figure 15:**
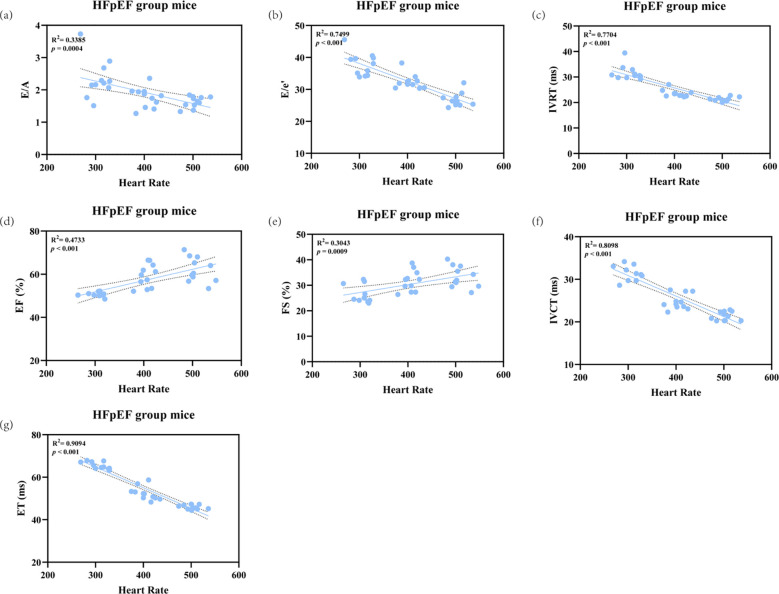
Correlation analysis in HFpEF group mice. **(a)** Correlation analysis of E/A ratio in HFpEF group mice; **(b)** Correlation analysis of E/e’ ratio in HFpEF group mice; **(c)** Correlation analysis of IVRT in HFpEF group mice; **(d)** Correlation analysis of LVEF values in HFpEF group mice; **(e)** Correlation analysis of FS values in HFpEF group mice; **(f)** Correlation analysis of IVCT in HFpEF group mice; **(g)** Correlation analysis of ET in HFpEF group mice.

As shown in **Figure 11**, the diastolic function indices E/A, E/e’, and IVRT decreased as the heart rate increased in normal rats. Among these, the regression models for E/e’ and IVRT demonstrated a strong linear fit with heart rate (R² = 0.7735 and R² = 0.6005, respectively). The systolic function indices EF and FS did not exhibit significant linear relationships. E/e’ showed a strong linear association with heart rate (R² = 0.7463) in the heart failure rat model group 7 days post-MI modeling. E/A exhibited a weaker linear trend with heart rate. IVCT, IVRT, and ET demonstrated strong linear dependence with heart rate. No linear relationships were observed for the systolic function indicators EF and FS (**Figure 12**). As shown in **Figure 13**, the E/A ratio in heart failure rats from the model group 28 days post-MI modeling showed a weak linear fit with heart rate (R² = 0.1976), while stronger linear regression models were observed for E/e’, IVCT, IVRT, and ET. No linear relationship was found for systolic function indicators EF and FS. In contrast, in normal mice, EF and FS values showed positive linear trends with heart rate, and E/e’, IVRT, and ET indicators exhibited strong linear fits with heart rate (**Figure 14**). Similarly, we observed significant linear dependence between these parameters and heart rate in the HFpEF model mice, with ET exhibiting the strongest fit (R² = 0.9094) (**Figure 15**).

## 4 Discussion

Heart rate is a core variable that fundamentally influences both hemodynamic and myocardial mechanical motion. Consequently, its variations can directly or indirectly alter multiple echocardiographic parameters, potentially leading to the misinterpretation of cardiac function outcomes in small animal models.

### 4.1 Influence of heart rate on systolic function

The assessment of left ventricular systolic function is regulated by the heart rate, primarily through myocardial contractility. LVEF is established as the gold standard for evaluating left ventricular systolic function. According to guidelines, LVEF ≤40%, LVEF ≥50%, and intermediate LVEF values denote HFrEF, HFpEF, and heart failure with mildly reduced EF, respectively (McDonagh et al., 2023).

In the present study, low heart rates managed under anesthetic manipulation did not significantly affect LVEF or FS values in rats. However, low heart rates controlled by isoflurane anesthesia had a greater impact on LVEF and FS values in mice than in rats. Their systolic function showed a marked decrease as the heart rate declined, particularly when it decreased to 250–350 bpm, where LVEF and FS values decreased significantly. Isoflurane reduces the heart rate by increasing the vagal tone. Since rats have a baseline heart rate substantially lower than mice (mice: approximately 500–700 bpm; rats: approximately 300–400 bpm), rats demonstrate a better tolerance of reduced vagal tone compared with mice, resulting in minimal impact on contractile function. Mice exhibit the opposite response; when heart rates decrease to 250–350 bpm, cardiac pumping efficiency is reduced, and systolic function is markedly suppressed. Relevant studies demonstrated that the cardiac structure of C57BL/6 mice is susceptible to the effects of isoflurane. An increase in the concentration of isoflurane results in thinning of the left ventricular wall of C57BL/6 mice and enlargement of the left ventricular cavity. Under 1% isoflurane anesthesia, they maintain good contractile function at normal levels. However, under 2% isoflurane anesthesia, the LVEF and left ventricular short-axis shortening fraction of C57BL/6 mice are decreased. Consequently, it is difficult to objectively evaluate the contractile function of the physiological state of the mice (Henriksnäs et al., 2005). Our results are consistent with these findings.

IVCT refers to the period from the onset of ventricular contraction to aortic valve opening, during which the ventricular pressure rises without ejection of blood. It reflects the isovolumetric phase of ventricular contraction, and prolongation indicates impaired contractile function. ET denotes the duration of ventricular ejection, spanning from aortic valve opening to closure. It reflects the efficiency of ventricular systolic ejection; a shortened ET indicates diminished systolic function. Measurement of both parameters is sensitive to heart rate variations. Consequently, their absolute values increase as the heart rate decreases regardless of a normal or pathological status.

### 4.2 Influence of heart rate on diastolic function

Left ventricular diastolic dysfunction is a core pathophysiological mechanism of heart failure, particularly in HFpEF. Its ultrasonic assessment relies heavily on measurements of ventricular filling patterns and myocardial relaxation velocity; both are closely related to the duration of the cardiac cycle and, thus, highly susceptible to heart rate influence. Our study revealed that reduced heart rate induces significant abnormalities in diastolic function-related parameters and impairs diastolic function in both rats and mice, regardless of normal or pathological conditions. The E/A ratio is a traditional indicator of diastolic function, defined as the ratio of the peak early diastolic mitral valve blood flow velocity wave E to the peak late diastolic atrial systolic blood flow velocity wave A. It is influenced by myocardial relaxation and ventricular stiffness, as well as by left atrial pressure. When the heart rate is significantly reduced or relaxation is markedly impaired, the atrium has more time to contract and pump blood into the ventricle, leading to an increase in A wave velocity (the filling velocity caused by atrial contraction) and a decrease in the E/A ratio. However, as left atrial pressure gradually increases or even becomes decompensated, the E/A ratio rises again with the progression of diastolic dysfunction, a phenomenon termed “pseudonormalization” (Oh et al., 1997; Nagueh et al., 2016).

The present study revealed that when the heart rate range decreased from 400–440 bpm to approximately 330–370 bpm, no significant differences in this ratio were observed within or between groups of normal and model rats. However, when the heart rate of normal rats decreased to approximately 250–290 bpm, the E/A ratio exhibited the aforementioned phenomenon, suggesting that diastolic function is severely suppressed in normal rats at low heart rates. Similarly, normal and HFpEF mice also demonstrated impaired cardiac diastolic function at low heart rates (250–350 bpm). Notably, this was not attributable to pathological myocardial injury in the animals. Research indicates that low heart rate is a mechanism increasing the E/A ratio (de Pontes et al., 1999). The significant prolongation of diastole alters ventricular filling patterns, enhancing early passive filling (E wave) while relatively diminishing late active atrial filling (A wave), thereby elevating the E/A ratio. Concurrently, the reduced time available for ventricular pressure decline results in physiologically prolonged IVRT. As demonstrated by our results, this occurs because the slowed ventricular pressure declines during prolonged diastole –induced by reduced heart rate – extends the interval between aortic valve closure and mitral valve opening. Conversely, it has been shown that high heart rates shorten IVRT (Wang et al., 2018). Consequently, ultrasound data obtained under these heart rate conditions cannot be objectively evaluated.

Our study revealed that partial fusion of the E/A peak in ultrasonic recordings occurred in rats when heart rates ranged 330–370 bpm. In mice, the probability of peak fusion increased at a heart rate range of 480–510 bpm. According to previous studies (Shang et al., 2000), consistent measurement remains highly challenging or impossible when E and A waves fuse. Consistent measurement of the E/A ratio in mouse hearts may only be feasible by artificially reducing the heart rate, such as through deeper anesthesia. However, this approach is linked to a risk of cardiac suppression, which is undesirable. Therefore, within heart rate ranges of 320–380 bpm (for rats) and 480–510 bpm (for mice), tissue Doppler imaging measurements are likely to encounter fused peaks, rendering E/A ratio measurement impossible. Therefore, controlling rat heart rates within 400–440 bpm and mouse heart rates within 450–480 or 510–550 bpm minimizes fusion peak occurrence without suppressing cardiac diastolic function, representing suitable measurement ranges.

The E/e’ ratio measured by tissue Doppler imaging is considered a core indicator for assessing left ventricular myocardial relaxation capacity, with minimal influence from preload (Mori et al., 2004; Oh et al., 2011). This ratio correlates with left ventricular filling pressure, which increases as diastolic dysfunction progresses. Clinically, an E/e’ ratio >15 is considered abnormal, indicating markedly elevated filling pressures. A ratio within the gray zone of 8 to 15 suggests potential diastolic dysfunction. However, given the substantial variations in heart rate and organ size across species, the applicability of commonly used echocardiographic parameters for assessing diastolic function in humans has not been extensively validated across various mouse pathophysiological models (Schnelle et al., 2018). In a cardiac rehabilitation study, exercise-induced increases in heart rate were associated with elevated e’ velocity and a significant reduction in the E/e’ ratio (Lee et al., 2021). We found that the E/e’ ratio in both normal and heart-failing rats and mice was sensitive to heart rate changes. Within each group, the E/e’ ratio showed a statistically significant increase as the heart rate decreased, with all intergroup comparisons yielding statistically significant results. Under physiological conditions, the prolonged diastole caused by low heart rate extends the pulmonary venous return time, resulting in the accumulation of more blood in the left atrium. This increases the pressure gradient between the left atrium and left ventricle, elevating the E wave. Pathological factors, such as MI-induced heart failure and structural remodeling in HFpEF, further elevate left atrial pressure. Additionally, impaired myocardial relaxation capacity, combined with the synergistic effects of elevated left atrial pressure driving high E waves and low e’ waves, contributes to relaxation dysfunction, further increasing the E/e’ ratio. Correlation analysis revealed that the E/e’ ratio showed a strong correlation with heart rate in normal rats, rats with heart failure 7 days post-MI, normal mice, and mice with HFpEF. The data showed that a high E/e’ combined with a high E/A ratio indicates severe diastolic dysfunction with elevated filling pressures and left atrial enlargement, denoting a poor prognosis (Oikawa et al., 2017). Considering the aforementioned understanding of the E/A ratio, ultrasound measurement of E/A and E/e’ ratios within the 250–350 bpm heart rate range may cause abnormal diastolic function indicators in normal animals and lead to false positives in model data interpretation. Heart rate control should be prioritized, or an assessment integrating multiple parameters of cardiac function should be employed under these conditions. A low e’/a’ ratio indicates poor myocardial diastolic function. According to the literature, this ratio is less frequently applied compared with E/A and E/e’, warranting further exploration. Based on measurements of systolic and diastolic function across three heart rate ranges in rats and mice, combined with consensus-recommended rodent parameters (Zacchigna et al., 2021), the practical heart rate measurement windows under isoflurane anesthesia in rats and mice are as follows. Firstly, rat systolic function remained essentially unchanged across all three heart rate ranges and showed no correlation with heart rate. However, for diastolic function, compared with the heart rate range of 420 ± 30 bpm, concurrent abnormalities in indicators such as E/e’ occurred at heart rates ranging 350 ± 30 and 270 ± 30 bpm, respectively, exceeding normal ranges and exhibiting significant correlation with heart rate. Therefore, it is recommended to maintain rat heart rates ≥390 bpm. Secondly, as heart rate decreases, contractile function measured by cardiac ultrasound in mice is significantly impaired. Additionally, multiple indicators of diastolic function, including E/e’, e’/a’ and IVRT, exhibit marked abnormalities due to the gradient induced decline in heart rate.

### 4.3 Advantages and limitations

In this study, we investigated changes in systolic and diastolic function across three heart rate gradient ranges during cardiac ultrasound in normal rats and mice, as well as in two disease models (HFrEF and HFpEF). We compared differences in cardiac function outcomes between normal and model groups under identical heart rate conditions, while analyzing the range of fusion peak occurrence. The findings indicate that, regardless of physiological or pathological status, ultrasound-monitored systolic function in rats is less affected by heart rate variations, whereas systolic function in mice declines with lower heart rates. Lower heart rates similarly induce diastolic dysfunction in both rats and mice. Therefore, during rodent cardiac ultrasound examination, maintaining a higher heart rate range (rats: 390–450 bpm; mice: 450–480 and 510–550 bpm) offers higher reproducibility and stability, better reflecting cardiac function changes in small animals.

However, this study also had limitations. Firstly, heart rate was controlled by titrating isoflurane concentrations. The observed differences in cardiac function across groups represent a combined effect of both heart rate reduction and varying depths of anesthesia, rather than the isolated effect of heart rate alone. Secondly, because all echocardiographic evaluations were performed under anesthesia, the recommended heart rate ranges are specific to these experimental conditions and may not fully represent physiological cardiac performance in conscious, natural states. Future studies comparing these findings with physiological reference values from conscious or lightly sedated animals are warranted. Finally, the investigation employed only two models; future research could utilize more commonly used models, such as the chronic stress overload model, while increasing the sample size. This would allow for the exploration of the correlation between heart rate variability and echocardiographic parameters in rats and mice, providing a reference basis for the accurate and reproducible quantification of left ventricular function under both healthy and pathological conditions.

## 5 Conclusions

The results of this study indicate that, regardless of physiological or pathological conditions, the systolic function of rats under ultrasound examination is minimally affected by heart rate changes. Conversely, the systolic function of mice declines as the heart rate decreases (<450 bpm). Both rats and mice exhibited significant susceptibility of their diastolic function to low heart rates, with rates <380/450 bpm in rats/mice, leading to diastolic dysfunction. It is noteworthy that E/A fusion peaks occur with a probability of 72.73% in rats and 81.82% in mice within the 330–370 bpm and 480–510 bpm ranges, respectively, affecting data measurement and interpretation. Therefore, at higher heart rates (rats: 390–450 bpm; mice: 450–480, 510–550 bpm), cardiac ultrasound data exhibit greater stability and reproducibility and minimize the occurrence of E/A fusion peaks. This range represents practical heart rate measurement window for small animal cardiac ultrasound examination under isoflurane anesthesia protocols. The present study provides scientific evidence for standardizing cardiac ultrasound examinations in rats and mice under varying heart rate conditions.

## Data Availability

The original contributions presented in the study are included in the article/supplementary material. Further inquiries can be directed to the corresponding author.
